# Isolation and partial characterization of Asian sea bass (*Lates calcarifer*) Vitellogenin

**DOI:** 10.1007/s10695-012-9690-5

**Published:** 2012-08-10

**Authors:** N. M. R. Fazielawanie, S. S. Siraj, S. A. Harmin, M. Y. Ina-Salwany

**Affiliations:** 1 Department of Aquaculture, Faculty of Agriculture, University Putra Malaysia (UPM), 43400 Serdang, Selangor Malaysia; 2 Centre for Land and Aquatic Technology, Faculty of Science and Biotechnology, University Industry Selangor (UNISEL), 45600 Batang Berjuntai, Selangor Malaysia

**Keywords:** Chromatography, *Lates calcarifer*, Phosphoglycolipoprotein, Vitellogenin, Western blot, Amino acid analysis

## Abstract

A study was conducted to isolate, partial characterize Asian sea bass (*Lates calcarifer*) vitellogenin (vtg). Two-year-old juvenile *L. calcarifer* (*n* = 10) were given three intraperitoneal injections of 17-β estradiol (E_2_) at a dose of 2 mg/kg body weight to induce vitellogenesis. Blood was collected 3 days after the last injection, and plasma was purified through gel filtration chromatography. A broad single symmetrical peak consisting of vtg molecule was produced. Protein concentration was 0.059 mg/ml as determined by Bradfrod assay using bovine serum albumin as a standard. The protein appeared as one circulating form in Native PAGE considering the dimeric form of putative vtg with molecular weight of 545 kDa. In SDS-PAGE under reducing conditions, two major bands appeared at 232.86 and 118.80 kDa and minor bands at 100.60, 85.80 and 39.92 kDa, respectively. The purified vtg was used to generate a polyclonal antibody, and the specificity of antibody was assessed by Western blot analysis. Two major bands were immunoreacted, but no cross-reactivity was observed with plasma from non-induced males. The protein was characterized as phosphoglycolipoprotein as it positively stained for the presence of lipid, phosphorus and carbohydrate using Sudan Black B, methyl green and periodic acid/Schiff reagent solution, respectively. The amino acid composition was analyzed by high sensitivity amino acid analysis that showed high percentage of non-polar amino acids (~48 %). The results suggest the potential utilization of vtg as a basis tool to further study about reproductive physiology of this important economical species.

## Introduction

*Lates calcarifer*, known as barramundi, Asian sea bass or locally called siakap, is native to coastal Australia, Southeast and Eastern Asia, and India (Luna 2008). This species are farmed in cages, as well as in fresh water and salt water ponds (Webster and Lim 2002). In recent years, sea bass has gained growing importance in aquaculture both as recreational and commercial fish, with a high and fairly stable price (Luna 2008). Sea bass production and consumption in Malaysia has increased dramatically over the years from 49, 586.19 (DOF 2005) to 194, 623.11 tonnes (DOF 2009). In spite of its economic importance, knowledge about its reproductive physiology is limited. In recent years, the reproductive biology of many fish species has been studied by analyzing the vitellogenin (vtg) levels in the blood plasma (Susca et al. 2001).

Vitellogenin is an egg yolk precursor protein that is synthesized in the liver under estrogen control and secreted into the bloodstream (Celius and Walther 1998; Prakash et al. 2007). This large protein has high molecular weight ranging from 250 to 600 kDa depending on fish species (Utarabhand and Bunlipatanon 1996). In teleost oviparous fish, vtg is served as a nutritional source for growing oocyte and developing embryo in matured female through a process called vitellogenesis (Romano et al. 2004). Following oocyte growth, vtg will enzymatically cleave into smaller yolk proteins namely lapidated lipovitellin, phosphorylated phosvitin and β-components (Zhang et al. 2011). Mananos et al. (1994) noted that the main components that contribute to the circulation of vtg molecule are lipids, carbohydrates, phosphorus and various ions such as calcium and iron. The vtg was present in adult vitellogenic females but absent in male as well as in immature females (Nath 1999; Fenske et al. 2001). In vertebrate, vtg gene is expressed in male and immatured female, but insufficient circulating estrogen is incapable to enhance the production of this protein (Palumbo et al. 2007). However, these organisms will synthesize the vtg if they are administered with synthetic estrogens, mainly 17-β estradiol (Leonardi et al. 2010; Boucard et al. 2008). Estradiol was reported to successfully induce the synthesis of vtg in many fish species (Utarabhand and Bunlipatanon 1996; Mendoza et al. 2011). The levels of vtg in fish indicate the maturing stage in female individual under natural condition (Matsubara et al. 1994). Knowledge of reproductive physiology including vitellogenesis is very important in managing fish broodstock for reproduction in most farmed animals including fish.

Previous studies reported that vtg has been isolated and purified in many fish species including Senegalese sole (*Solea senegalensis*) (Guzman et al. 2008), grouper (*Ephinephelus malabaricus*) (Utarabhand and Bunlipatanon 1996), California halibut (*Paralichthys californicus*) (Palumbo et al. 2007), Chilean flounder (*Paralichthys adpersus*) (Leonardi et al. 2010) and sea bass (*Dicentrarchus labrax* L.) (Mananos et al. 1994) by using double chromatography, ion exchange followed by gel filtration chromatography. However, Watts et al. (2003) and Mendoza et al. (2011) have proven that gel filtration alone using Sepachryl 16/60 HR-300 column was able to completely isolate vtg in three teleost species.

Vitellogenin has never been purified and characterized in *L. calcarifer,* and knowledge about its reproduction and vitellogenesis is needed. A better understanding of vitellogenesis is very important for farm management as well as to determine the maturity status of this economically important species (Utarabhand and Bunlipatanon 1996). Thus, the aim of this study was to induce, purify and characterize the vtg from E_2_-treated juvenile *L. calcarifer.*

## Materials and methods

### Experimental animals

Sampling was done at Center of Marine Science, Universiti Putra Malaysia, Port Dickson, Malaysia. Two-year-old juvenile Asian sea bass (*L. calcarifer*) (*n* = 10), ranging in weight from 1.5 ± 0.5 kg, were obtained from commercial supplier, while matured males and vitellogenic females, weighing around 4.0–6.3 kg, were obtained from Fisheries Research Institute, Tanjung Demong, Terengganu, Malaysia. They were maintained for 2 weeks in 10-tonne tank with filtered, flow-through seawater at salinity of 29 ± 1 ppm, a temperature of 21 ± 2 °C, pH of 6.0–6.5 and supplied with dissolved oxygen (5.4 mg/ml). Fishes were tagged using microchips for further identification and fed daily at satiation with chopped fresh fish.

### Induction of vtg

Each juvenile fish was injected with 17-β estradiol, E_2_ (Nacalai Tesque, Japan) to induce the vitellogenesis. They received a total of three intraperitoneal injections (i.p.) (dose of 2 mg E_2_/kg fish body weight), given every 2 days. Estradiol was dissolved in ethanol and 0.9 % NaCl solution (1:9, v/v) as a carrier. Control fishes (*n* = 6) were injected with saline only, and no foods were administered during the experiment.

### Plasma preparation

Three days following the last injection, four millilitres of blood was collected from the caudal vein of each fish using heparinized syringe containing phenylmethylsolfonyl fluoride, PMSF (Roche, Germany) (100 μl, 1 mM). The blood was maintained on ice, allowed to clot at 4º C for 1 h and centrifuged at 8,000 rpm for 10 min at 5 °C (Braathen et al. 2009). The supernatant (plasma) was withdrawn and immediately stored in the presence of PMSF to avoid proteolysis of vtg at a ratio of 2: 1 v/v (plasma:PMSF) (Watts et al. 2003). It was stored at −80 °C prior to vitellogenic purification (Parks et al. 1999). At the same time, blood was collected from control group (vitellogenic females and non-induced males).

### Purification of vtg

Plasma vtg was purified using gel filtration chromatography (Akta Prime) performed at room temperature according to Watts et al. (2003) with modifications. One millilitre of plasma from estradiol treated juvenile *L. calcarifer* was loaded into prepacked Sepachryl HR-300 column (HiPrep 16/60) (GE Healthcare Bio-Science, Uppsala, Sweden). The samples were eluted with 0.05 M Tris–HCl pH 8.0 (Nacalai tesque, Japan) at a flow rate of 0.4 ml/min. The elution profile was monitored at 280 nm, and the fractions containing vtg peak were collected at a final volume of 5 ml. The purified vtg consisting peak was pooled and directly concentrated using Vivaspin centrifuge tube (30 kDa Molecular weight cut-off, GE Healthcare Bio-Science, Uppsala, Sweden) at 4 °C, 10,000 rpm for 10 min. It was stored at −20 °C in aliquots before subjected to Native PAGE and SDS-PAGE analysis. The purified vtg was used as antigen for antibody production against vtg in rabbits. The protein concentration was determined by Bradford assay using bovine serum albumin (BSA) (Sigma Diagnostics, USA) as standard.

### Characterization of purified vtg

#### Native PAGE

To determine the purity and molecular weight of circulating form of *L. calcarifer* vtg, purified plasma was subjected to Native gradient PAGE (4–8 % separating gel solution) (Sun and Zhang 2001) with constant current of 100 mA for 4 h on ice in an electrophoretic buffer (0.025 M Tris and 0.192 M glycine. The molecular weight was determined by comparing with Native markers (Serva, Heidelberg, Germany). Separated protein was identified as phosphoglycolipoprotein (vtg) after staining with Sudan Black B, periodic acid/Schiff reagent solution (PAS) and methyl blue (Nacalai tesque, Japan) to determine the presence of lipid, carbohydrate and phosphorus, respectively.

#### Sodium dodecyl sulfate polyacrylamide gel electrophoresis (SDS-PAGE)

To determine the molecular weight of vtg subunits, the purified plasma vtg was electrophoresed (SDS-PAGE) under denaturing conditions. It was performed on 7.5 % separating gel and 4 % stacking gel. Prior to application on the gel, the purified vtg were diluted in SDS sample buffer (125 mM Tris–HCl, 10 % SDS, 20 % v/v glycerol, 5 % v/v β-mercaptoethanol, 0.02 % bromophenol blue) (15 μg/ml) and boiled for 5 min. Electrophoresis was run on ice in a buffer (50 mM Tris, 192 mM glycine and 0.1 % SDS) at a constant current of 100 mA, 50 V for 5 h and immediately subjected to Western blot analysis. The results obtained were viewed using gel imager (Alpha Innotech, Cell Bioscience, California). The molecular weights of purified vtg were estimated by comparing with molecular weight protein markers (Fermentas, USA).

### Production of polyclonal antibodies

Specific antiserum against *L. calcarifer* vtg was raised in seven-month-old (~3 kg) female New Zealand white rabbits (*n* = 4). Rabbits were initially injected with freshly purified vtg (0.059 mg/ml protein) emulsified in Freund’s Complete Adjuvant (CFA, Calbiochem) (1:1 v/v, 1 ml) and then boosted up by four additional injections of 0.02 mg/ml protein emulsified in Freund’s Incomplete Adjuvant (IFA, Calbiochem) (1:1 v/v, 1 ml) at multiple subcutaneous and intradermal sites. A total of five injections were given at weeks 1, 3, 5, 7 and 9. Blood (~2.0 ml) was collected at weeks 4, 6 and 10, and the serum was assayed for reactivity toward vtg by screening ELISA. When antibody titer was sufficient, blood (~10 ml) was withdrawn from the ear artery and allowed to clot overnight at 4 °C (Smith and Benfey 2002). Serum was separated by centrifuging the blood at 12,000×*g* at 4 °C for 10 min and then stored at −80 °C in small aliquots. For negative antibody control, the rabbits were bled before immunization (pre-immune serum).

### Immunoblotting (Western blot)

The purity of antigen and specificity of antibodies were tested by using Western blot analysis. The proteins separated by Native PAGE and SDS-PAGE were electro-blotted onto polyvinylidene fluoride, PVDF membrane (Immobilon-P, Millipore). The unstained gels were soaked in transfer buffer (48 mM Tris, 39 mM glycine, 20 % methanol, pH 9.2) for 15 min, and the blot was run at constant current of 300 mA, 50 V for 2 h on ice cold using Bio-Rad Trans Blot Cell. Following the transfer, the membranes were stained with Coomassie brilliant blue R-250 (0.025 % Coomassie Blue R-250, 40 % methanol, 7 % acetic acid) (Hercules, Canada). PVDF membranes were blocked by incubating in TBS (50 mM Tris, 150 mM NaCl, pH 7.5) containing 3 % bovine serum albumin (BSA) for 2 h to prevent non-specific binding sites. For immunochemical detection, the membranes were then incubated with primary antibody (anti-Vtg in rabbits) at a dilution of 1:1,500 in blocking buffer. Bound antibodies were detected by incubating with secondary antibody, HRP (Horse-Reddish Peroxidase, conjugated goat anti-rabbit IgG) (Nacalai tesque, Japan) at a dilution of 1:2,000 in blocking buffer for 2 h at room temperature. For visualization, membrane was incubated with substrate solution, Opti-4CN™ Substrate Kit (Bio-Rad, Hercules, CA) for 30 min to reveal the location of vtg. The membranes were washed with TBST [50 mM Tris, 150 mM NaCl, pH 7.5 containing 0.05 % Tween-20 (v/v)] three times for 15 min after each incubation step. The developed membranes were photographed using gel imager.

### Amino acid analysis

For analysis of amino acid composition, the purified vtg (200 μl) was analyzed by high sensitivity amino acid analysis (AAA). The sample was lyophilized and resuspended in a solution of 20 % acetonitrile (ACN) and 0.1 % trifluoroacetic acid (TFA). Sample underwent 24-h gas-phase hydrolysis with 6 N HCl at 110 °C. After hydrolysis, all amino acids were analyzed using high performance liquid chromatography (HPLC) system (Waters AccQTag Ultra) chemistry. The analyses were carried out in duplicate, and results were expressed as means. Amino Acid Standard H (Thermo Scientific Pierce) was used as calibration standard for HPLC analysis.

## Results

### Purification of vtg

**Fig. 1:**
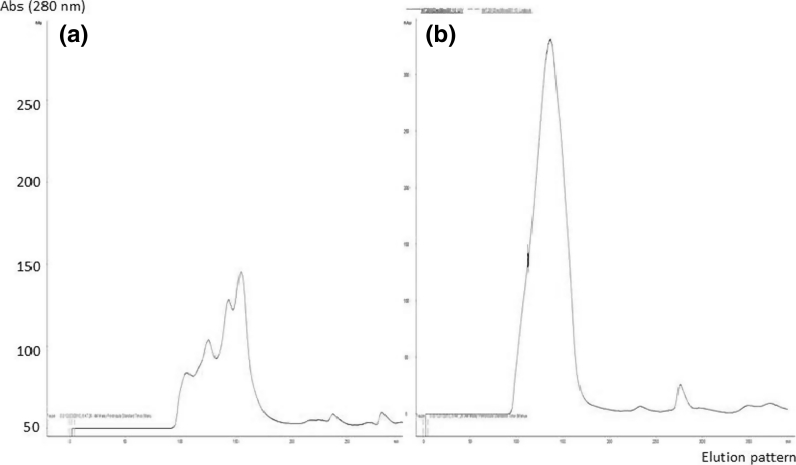
Gel filtration chromatography from plasma of juvenile *L. calcarifer* before (**a**) and after treated with 17-β estradiol (**b**) using Sepachryl (16/60) HR 300 column (GE healthcare Bioscience, Uppsala, Sweden)

Vitellogenin was purified from plasma of E_2_-treated juvenile *L. calcarifer* by gel filtration chromatography using Sepachryl HR-300 column (GE Healthcare Bio-Science). Figure 1 shows the elutionlysine were present at high abundance pattern of juvenile *L. calcarifer* before (a) and after treatment with 17-β estradiol (b). A single symmetrical peak was obtained from purification of E_2_-treated fish indicating the presence of vtg molecule. This peak was absence in chromatographic profile from non-induced. Absorbance was read at OD 280 nm. The protein concentration of purified plasma was 0.059 mg/ml as determined by Bradford assay.

### Characterization of purified vtg

**Fig. 2:**
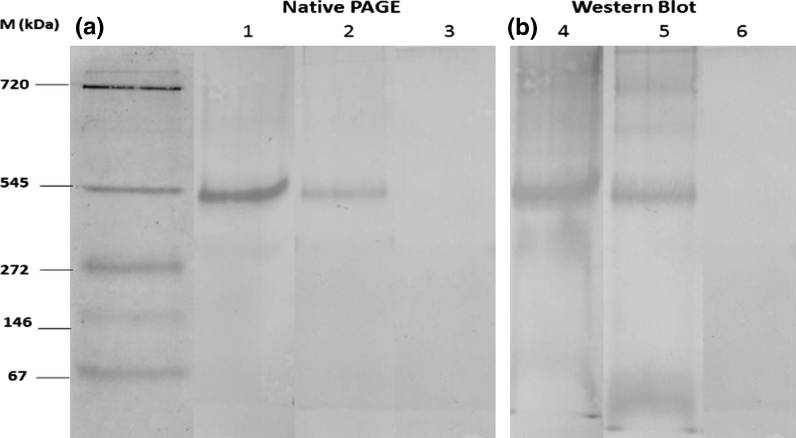
**a**, **b** Native PAGE electrophoretic pattern (**a**) and subsequent western blot analysis (**b** *Lanes 1* and *4* purified plasma from 17-β estradiol treated fish, *lanes 2* and *5* plasma from natural vitellogenic females, *lanes 3* and *6* plasma from non-induced male. The proteins were stained with Coomassie Brilliant Blue R-250 (Bio-Rad, Hercules, Canada)

Under native gradient PAGE analysis, vtg appeared as one dimeric circulating form in the E_2_-treated fish (Fig. 2a, lane 1) with molecular weight of 545 kDa. The same band position was also shown from plasma of natural vitellogenic females (Fig. 2a, lane 2). No protein was detected from the plasma of non-induced male (Fig. 2a, lane 3). The corresponding Western blot analysis using anti-Vtg polyclonal antibody showed strong reactivity with E_2_-treated *L. calcarifer* and vitellogenic females (Fig. 2b, lanes 4 and 5). There was no cross-reactivity with the plasma from the control male (Fig. 2b, lane 6). Native polyacrylamide gel electrophoresis confirmed that the purified vtg was pure and free from contaminants (Lomax et al. 1998).

**Fig. 3:**
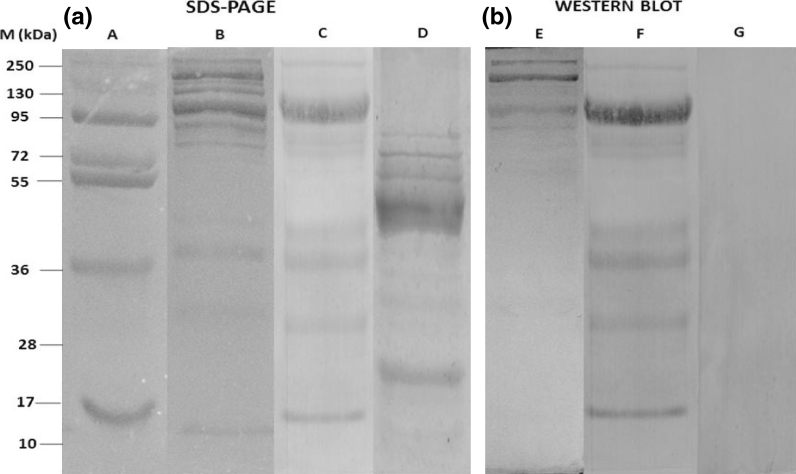
**a**, **b** SDS-PAGE (0.1 % SDS) (*A*) and corresponding Western blot analysis (*B*), stained with Coomassie Brilliant Blue. *Lane A* molecular weight markers (Fermentas, USA); *lane B* and *E* purified vtg; *lanes C* and *F* vitellogenic females; *lanes D* and *G* male plasma

Electrophoresis of the purified plasma from E_2_-treated *L. calcarifer*, vitellogenic females and untreated males under reducing conditions is shown in Fig. 3. The molecular weight of purified vtg from peak B resulted in five subunits with two major bands (232.86 and 118.80 kDa) and other minor bands (100.60, 85.80 and 39.92 kDa; lane B). The major bands were initially not appeared in the control male plasma (lane D), but shown in those induced by 17-β estradiol (lane B). The same band position occurred in vitellogenic female as in the E_2_-treated fish at molecular weight of 118.80 kDa (lane C). The corresponding Western blot analysis in SDS-PAGE showed that the specific Abvtg polyclonal antibody was immunoreacted with plasma from vitellogenic females (lane F) and only reacted with two major bands of E_2_-treated *L. calcarifer* (lane E), but did not recognize any bands in non-induced male (lane G). This indicated that vtg was specific with Abvtg, which did not react with any non-vitellogenic proteins.

**Fig. 4:**
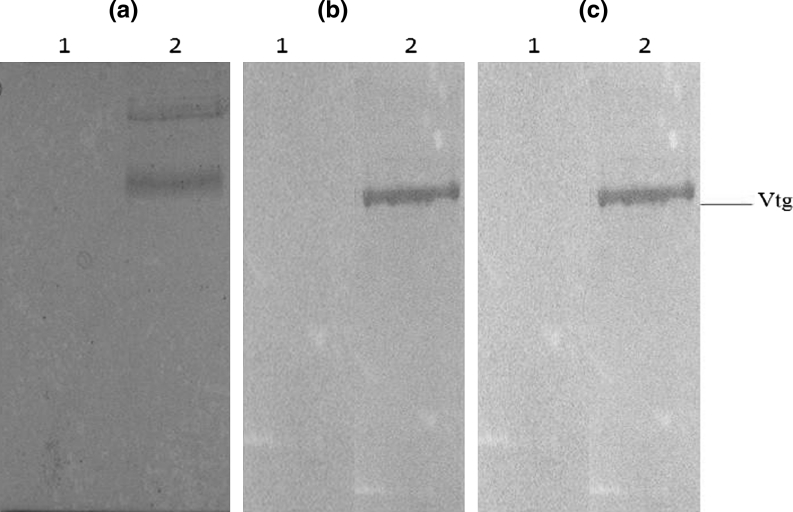
**a**–**c** Native PAGE for determination of lipid phosphorus and carbohydrate components in vtg of *L. calcarifer*. *Lane 1* male plasma, *lane 2* E_2_-treated plasma. Gels were stained with Sudan black B (**a**), methyl green (**b**) and Periodic acid/Schiff reagent solution (PAS) (**c**), respectively

On the other hand, the protein was positively stained with Sudan black B, methyl green and periodic acid/Schiffs reagent solution. This indicated the presence of lipid, phosphorus and carbohydrate in vtg molecule (Fig. 4).

### Amino acid analysis

Amino acid composition of E_2_-treated juvenile *L. calcarifer* is shown in Table 1. It contained high percentage of non-polar amino acids (glycine, alanine, proline, valine, isoleucine and leucine: ~48 %). Alanine was most abundant, comprising approximately 10.1 % of total amino acids. Charged and non-polar composed of approximately 52 % of total amino acids. Both serine and lysine were present at high abundance 8.3 and 6.7 %, respectively.

**Table 1:** Total amino acid composition of vtg in Asian sea bass in comparison with other fish species by percentage of moles

Amino acid	Percentage of total amino acids				
	Asian sea bass (the present study)	Fathead minnow (Parks et al. 1999)	Carp (Tyler and Sumpter 1990)	Grouper (Utarabhand and Bunlipatanon 1996)	Striped bass (Tao et al. 1993)
Aspartic acid	8.2	7.1	6.7	6.5	7.6
Threonine	7.9	5.6	5.4	4.7	5.6
Serine	8.3	6.3	7.6	5.5	7.2
Proline	5.0	6.1	5.9	4.0	4.3
Glutamic acid	11.0	12.3	11.8	12.0	11.9
Glycine	7.3	4.6	5.1	5.5	4.2
Alanine	10.1	12.0	12.6	10.2	12.0
Valine	8.1	7.9	6.3	7.6	7.9
Methionine	2.1	1.9	1.9	2.0	1.9
Isoleucine	4.6	6.7	5.4	6.5	7.0
Leucine	7.9	10.8	10.5	10.3	10.8
Tyrosine	3.9	2.3	2.8	2.6	3.4
Phenylalanine	3.9	2.7	2.8	2.8	3.4
Lysine	6.7	6.6	6.3	7.0	7.3
Histidine	2.1	2.2	3.4	2.4	3.1
Arginine	4.1	4.4	5.0	7.9	4.9
Cystine/2	ND	0.0	0.1	ND	1.0

*ND* Not determine

## Discussion

The induction of 17-β estradiol in juvenile *L. calcarifer* resulted in the production of high-molecular-weight protein (232.18 and 118.80 kDa). The same amount of high molecular weight protein appeared in vitellogenic female that is identified as vitellogenin (vtg). It is well established that vtg was present abundantly in blood plasma of natural vitellogenic females. However, it can be induced in males and juvenile fish by treating with estrogen hormone (Smith and Benfey 2002; Rankouhi et al. 2001).

There are a few steps involved in the isolation and separation of vtg molecules. In the present study, the vtg was isolated and purified by using gel filtration chromatography of Sepachryl HR-300 column. Chromatography was the best method to isolate vtg from blood plasma of induced fish as successfully proven by several studies (Watts et al. 2003; Roy et al. 2004; Palumbo et al. 2007; Braathen et al. 2009; Maltais and Roy 2009; Leonardi et al. 2010). A broad, single symmetrical peak (Fig. 1b) from purification of E_2_-treated juvenile *L. calcarifer* obtained in this study indicated that the peak was E_2_-inducible, and there was no sign of degradation occurred during the isolation process (Guzman et al. 2008). Norberg (1995) also obtained a single molecule after vtg purification in Atlantic halibut (*Hippoglossus hippoglossus*). Roy et al. (2004) suggested that the best condition to isolate and purify vtg was at lower temperature (4º C) so as to preserve the stability of the protein. However, in this present study, purification process was carried out at room temperature. Hence, to avoid degradations, it was run in the presence of protease inhibitor (PMSF), which contributes to the isolation of single vtg molecule (Mosconi et al. 1998). Similar result was also observed for vtg in carp, *Cyprinus carpio* and perch, *Perca fluviatilis* (Hennies et al. 2003). However, purification analysis alone cannot determine that the isolated protein was vtg molecule. It needs to be confirmed by cross-reactivity of anti-vtg raised in New Zealand white rabbits in immunoblotting analysis (Norberg 1995).

There are a few evidences proven that the purified protein was phosphoglycolipoprotein (vtg). Firstly, the plasma protein levels in induced *L. calcarifer* were increased compared to non-induced (0.008–0.059 mg/ml, data not shown). This is in agreement with the finding by Mananos et al. (1994). Secondly, the specificity of antiserum confirmed by Western blot analysis showed that polyclonal antibody have strong reactivity in estrogen exposed juvenile *L. calcarifer* as well as vitellogenic females but failed to show any reaction with plasma from non-induced males. This indicates that the polyclonal antibody was specific, which failed to react with any non-vitellogenic proteins. Similar result was also obtained by Tao et al. (1993) in striped bass (*Morone saxatilis*) and Zhang et al. (2011) in Amur sturgeon (*Acipenser schrenckii*). Thirdly, in native gradient PAGE (samples were run without SDS and β-mercaptoethanol), the protein was positively stained for the presence of phosphorus, carbohydrate and lipid using methyl blue, periodic acid/Schiff’s reagent solution (PAS) and Sudan black B as previously reported (Egito et al. 2001; Sun and Zhang 2001; Roy et al. 2004). Fish vtg was generally known to have high phosphorus and lipid contents that normally present as phospholipid and triglycerides (Tao et al. 1993). Fourthly, the amino acid composition of *L. calcarifer* vtg was very similar to vtg as previously noted from other fish species (Utarabhand and Bunlipatanon 1996; Tao et al. 1993; Parks et al. 1999; Tyler and Sumpter 1990). In the present study, high percentage of non-polar amino acids (glycine, alanine, proline, valine, isoleucine, leucine, ~48 %) was found which contributed to its lipoprotein function for transportation of endogenous lipid (Parks et al. 1999; Tao et al. 1993; Kera et al. 2000).

The analysis of vtg on native gradient PAGE resulted only one band (Fig. 2) confirmed that the isolated protein was free from contaminants. Hence, it is proved that gel filtration alone was able to completely isolated vtg in *L. calcarifer*. Palumbo et al. (2007) showed that the analysis of vtg by Native PAGE also resulted one circulating band interpreted that the band was dimeric form of putative vtg, which was similar to the present study. Sun and Zhang (2001) reported that the vtg from fish, amphibians and birds, nematodes and arthropods also circulate as dimeric form. Native PAGE analysis resulted an apparent molecular weight 545 kDa of *L. calcarifer* vtg, which falls within the range of vtg dimeric form as shown in other fish species: 525 and 260 kDa in grouper (Utarabhand and Bunlipatanon 1996), 490 kDa in carp (Fukada et al. 2003), 454 kDa in sea bass (Mananos et al. 1994), 330 kDa in female Bluefin tuna (Susca et al. 2001) and 390 kDa in teleost sp. (Larsson et al. 1994).

The molecular weight of vtg (232.86 and 118.80 kDa) in Asian sea bass (*L. calcarifer*) was greater than other fish species: that is 205 kDa for Amur Sturgeon *Acipenser schrenckii* (Zhang et al. 2011), 183 kDa for Gag *Mycteroperca microlepis* (Heppel and Sullivan 1999), 220 kDa for Medaka *Oryzias latipes* (Shimizu et al. 2002), 150 kDa for carp *Cyprinus carpio* (Fukada et al. 2003), 172 kDa for *S. senegalensis* (Guzman et al. 2008) and 180 kDa for sea bass *D. labrax* (Mananos et al. 1994). The presence of minor bands represents the monomer form of vtg molecule probably due to the use of sodium dodecyl sulfate (SDS) and β-mercaptoethanol, which contributes to the degradation fragments of putative vtg and similar finding was noted by Bon et al. (1997). The difference in molecular weight of vtg in each species indicated the different structure of vtg molecule.

Previous study reported that vtg was unstable and easily degraded due to the storage and handling of plasma sample (Norberg 1995; Roy et al. 2004). Hence, in this study, plasma was stored in aliquots and purified in the presence of phenylmethylsulfonyl fluoride (PMSF) in a ratio of 2:1 v/v to prevent proteolysis of this labile protein (Watts et al. 2003).

## Conclusion

In conclusion, the present study was successfully isolated and partial characterized vitellogenin in induced juvenile *L. calcarifer* by using gel electrophoresis, Western blotting and amino acid analysis. Further analyses of vtg levels using Abvtg proposed as indicator of maturing female stage for managing fish broodstock in captivity.
